# Fragile Gene *WWOX* Guides *TFAP2A*/*TFAP2C*-Dependent Actions Against Tumor Progression in Grade II Bladder Cancer

**DOI:** 10.3389/fonc.2021.621060

**Published:** 2021-02-25

**Authors:** Damian Kołat, Żaneta Kałuzińska, Andrzej K. Bednarek, Elżbieta Płuciennik

**Affiliations:** Department of Molecular Carcinogenesis, Medical University of Lodz, Łódź, Poland

**Keywords:** bladder cancer, WWOX, TFAP2A, TFAP2C, AP-2α, AP-2γ

## Abstract

**Introduction:**

The presence of common fragile sites is associated with no-accidental chromosomal instability which occurs prior to carcinogenesis. The *WWOX* gene spans the second most active fragile site: FRA16D. Chromosomal breakage at this site is more common in bladder cancer patients who are tobacco smokers which suggests the importance of *WWOX* gene loss regarding bladder carcinogenesis. Tryptophan domains of WWOX are known to recognize motifs of other proteins such as AP-2α and AP-2γ allowing protein-protein interactions. While the roles of both AP-2 transcription factors are important for bladder carcinogenesis, their nature is different. Based on the literature, AP-2γ appears to be oncogenic, whereas AP-2α mainly exhibits tumor suppressor character. Presumably, the interaction between WWOX and both transcription factors regulates thousands of genes, hence the aim of the present study was to determine WWOX, AP-2α, and AP-2γ function in modulating biological processes of bladder cancer.

**Methods:**

RT-112 cell line (grade II bladder cancer) was subjected to two stable lentiviral transductions. Overall, this resulted in six variants to investigate distinct WWOX, AP-2α, or AP-2γ function as well as WWOX in collaboration with a particular transcription factor. Cellular models were examined with immunocytochemical staining and in terms of differences in biological processes using assays investigating cell viability, proliferation, apoptosis, adhesion, clonogenicity, migration, activity of metalloproteinases and 3D culture growth.

**Results:**

WWOX overexpression increased apoptosis but decreased cell viability, migration and large spatial colonies. AP-2α overexpression decreased tumor cell viability, migratory potential, matrix metalloproteinase-2 activity and clonogenicity. AP-2γ overexpression decreased matrix metalloproteinase-2 activity but increased wound healing, adhesion, clonogenicity and spatial colony formation. WWOX and AP-2α overexpression induced apoptosis but decreased cell viability, adhesion, matrix metalloproteinase-2 activity, overall number of cultured colonies and migration rate. WWOX and AP-2γ overexpression decreased tumor cell viability, proliferation potential, adhesion, clonogenicity and the ability to create spatial structures, but also increased apoptosis or migration rate.

**Conclusion:**

Co-overexpression of WWOX with AP-2α or WWOX with AP-2γ resulted in a net anti-tumor effect. However, considering this research findings and the difference between AP-2α and AP-2γ, we suggest that this similarity is due to a divergent behavior of WWOX.

## Introduction

Common fragile sites (CFSs) are chromosomal loci appearing as decondensations, gaps or breaks on metaphase chromosomes due to DNA replication failure in late S-phase/G2-phase or as damage persistent at mitosis (1, 2). Their no-accidental instability is frequently an initial step of tumor suppressor gene loss or oncogene amplification (3, 4), indicating them as preferential hotspots facilitating carcinogenesis and further cancer rearrangements. Three of the most active regions with such susceptibility are FRA3B, FRA16B, and FRA6E containing Fragile Histidine Triad Diadenosine Triphosphatase (*FHIT*), WW Domain Containing Oxidoreductase (*WWOX*) and Parkinson disease-2 (*PARK2*) genes, respectively (5, 6). Of these, *WWOX* is situated in direct proximity to the chromosomal locus 16q24 whose allelic loss is implicated in the tumor progression of 20% to 45% of bladder cancers (BLCAs) (7). The importance of this gene in terms of bladder cancer carcinogenesis is supported by the fact that FRA16D chromosomal breakage is more common in oncological patients who are tobacco smokers (8). The *WWOX* gene encodes the 46kDa oxidoreductase protein which contains specific domains (9); these include two WW domains on the N-terminal site and an SDR domain located centrally (10). WW domains are known to play role in protein-protein interactions because their tryptophans can recognize proline-rich (PPxY) motifs in other proteins such as the Activating enhancer-binding Protein 2α (AP-2α) and AP-2γ transcription factors (11). These are not without significance in BLCA since Yamashita *et al.* has proposed both factors as mediators of basal-squamous transition (12), thus potentiating the aggressive form of bladder cancer. However, while the role of the Transcription Factor AP-2 Gamma (*TFAP2C*) gene (encoding AP-2γ) in bladder cancer and various other cancer types currently appears unequivocal (13), the role(s) of the *TFAP2A* gene (encoding AP-2α) is uncertain. In contrast to findings of Yamashita *et al.*, high expression of AP-2α was also found to be associated with longer progression-free survival (PFS) and overall survival (OS) in cisplatin-treated advanced bladder cancer (14). In addition, knockdown of AP-2α-encoding gene intensified cancer proliferation and decreased chemotherapy-induced cell death, confirming its ameliorating effect on BLCA carcinogenesis (14). This can be supported by distinct study which revealed particular cross-links between AP-2α and miR-193a-5p (15). Zhou *et al.* argued that cisplatin resistance in UM-UC-3 bladder cancer cell line is induced by miRNA that diminished AP-2α expression (15), which highlights the necessity to identify the reasons of these discrepancies.

As the interaction between WWOX protein and transcription factors presumably entails the regulation of thousands of genes (thus affecting signaling pathways and further on biological processes), the aim of the present study was to determine the function of the genes encoding WWOX, AP-2α and AP-2γ in modulating the biological processes of bladder cancer. Due to the non-classical nature of at least two aforementioned genes, it was also decided to examine their collaborative functionality using cellular models with overexpression of either genes encoding WWOX and AP-2α or WWOX and AP-2γ.

## Materials and Methods

### Cell Line and Culture Conditions

The urinary bladder cancer cell line RT-112 was purchased from Deutsche Sammlung von Mikroorganismen und Zellkulturen (DSMZ, Germany). This cell line was cultured in RPMI-1640 medium supplemented with 10% heat-inactivated FBS, 1% Antibiotic-Antimycotic (100 units/ml of penicillin, 100 µg/ml of streptomycin, and 0.25 µg/ml of Fungizone) and 1% L-glutamine (Thermo Fisher Scientific, Netherlands). Cells were incubated at 37°C in a humidified atmosphere of 5% CO_2_.

### Stable Transductions

To overexpress the *WWOX* gene, the GIPZ™ lentiviral system (pLenti-GIII-CMV-GFP-2A-Puro) was used with Puro-Blank Lentivirus as a control (Applied Biological Materials Inc., Canada). Transduction was performed in starving medium containing 8 µg/ml polybrene (Merck Life Sciences Sigma Aldrich, Germany) and lentiviral particles at MOI=3. Viral medium was changed into full medium after 24** h** of incubation. Antibiotic-based clone selection was performed 72** h** later with the use of 1 µg/ml puromycin (Merck Life Sciences Sigma Aldrich, Germany).

The obtained variants mentioned above i.e. with overexpressed WWOX or without (control), were further subjected to a second transduction. To overexpress AP-2α and AP-2γ, the Lentiviral system (pLKO-Neo-CMV) was used with Puro-Blank Lentivirus as a control (Merck Life Sciences Sigma Aldrich, Germany). Transduction was performed in starving medium containing 8 µg/ml polybrene and lentiviral particles at MOI=3. After 24** h**, the starving medium was replaced by full medium and selection was continued on with 400 μg/ml G418 (Merck Life Sciences Sigma Aldrich, Germany) for three weeks.

Subsequent transduction was performed to overexpress WWOX and one of the following transcription factors: AP-2α or AP-2γ. Overall, stable transductions resulted in the production of the following variants for RT-112 cell line (with abbreviations in brackets):

Variants with an appropriate level of WWOX and AP-2α expression:

- Control/control (K/K); Control/AP-2α↑ (K/A); WWOX↑/control (W/K); WWOX↑/AP-2α↑ (W/A)
- Variants with an appropriate level of WWOX and AP-2γ expression:
- Control/control (K/K); Control/AP-2γ↑ (K/C); WWOX↑/control (W/K); WWOX↑/AP-2γ↑ (W/C)

Depending on the variant considered, comparisons were made against the appropriate control that was determined during first stable transduction (**Supplementary Figure 1**). Variants with overexpression of AP-2α or AP-2γ alone (K/A or K/C, respectively) were compared to K/K. Variants with both WWOX and AP-2α or AP-2γ overexpression (W/A or W/C) were compared with W/K. The variant with separate WWOX overexpression (W/K) was compared to K/K.

### Western Blot

All proteins were isolated using RIPA Lysis Buffer supplemented with sodium orthovanadate, phosphatase inhibitor cocktail and phenylmethylsulfonyl fluoride (PMSF) (Santa Cruz Biotechnology Inc., Dallas, TX, USA). The assay was performed as previously described (16). To confirm successful transduction, the following primary antibodies were used: 1:1,000 anti-human WWOX (PA5-29701, Thermo Fisher Scientific, Netherlands), 1:1,000 anti-human AP-2γ (GTX134259, GeneTex Inc., USA) and 1:1,000 anti-human AP-2α (GTX113564, GeneTex Inc., USA). The band intensity of each protein was evaluated using ImageJ analysis software (17) and normalized to GAPDH (sc-59540, Santa Cruz Biotechnology Inc., Dallas, TX, USA). The assay was performed in technical triplicate or duplicate for first or second stable transduction, respectively.

### Immunocytochemistry

The cells were fixed with ice cold ethanol:acetic acid (95:5) solution for 10** min**. After a double wash with D-PBS, non-specific antibody binding was blocked with blocking buffer (2% BSA, 5% donkey serum, and 0.1% Triton-X100). Following this, the cells were incubated with the primary antibodies in 1:100 solution in 5% donkey serum at 4°C, overnight. The catalog numbers were PA5-29701 (anti–WWOX), MA5-14856 (anti–AP-2α), PA5-17330 (anti–AP-2γ), supplied by Thermo Fisher Scientific, Netherlands. After double washing with D-PBS, the cells were incubated 1 h with secondary antibodies (donkey, Alexa Fluor 594, catalog number: A21207), diluted 1:1,000. The nuclei were counterstained with ProLong**™** Gold antifade reagent with DAPI (Thermo Fisher Scientific, Netherlands), the cells were then imaged at 40x magnification, 0.75 numerical aperture using Eclipse Ci-S (Nikon). The assay was performed in biological triplicate for each cell variant.

### Triplex Assay for Assessment of Redox Potential, Proliferation and Apoptosis

Three cellular assays were performed simultaneously on a single plate: these compared the differences in apoptosis, cell viability and proliferation between the examined variants. Cell line variants were seeded on a 96-well plate at a concentration of 1.5 × 10^4^ cells per well. After 24** h** of incubation, medium was changed into 90 µl starving medium with 10 µl 5-bromo-2′-deoxyuridine (BrdU) and the cells were incubated for the next 24** h**. Next day, 10 µl of PrestoBlue reagent (Thermo Fisher Scientific, Netherlands) was added to the wells and fluorescence signals (excitation of 550nm and emission of 590 nm) with 10** min** intervals were measured. As controls, we also used wells on plate without cells and reagent. Proliferation was detected using BrdU incorporation (DELFIA**^®^** Cell Proliferation Kit; PerkinElmer, Canada) and apoptosis based on TUNEL assay (DELFIA**^®^** DNA Fragmentation Assay; PerkinElmer, Canada). The procedure was conducted according to the manufacturer**’**s protocol. The fluorescence signal was detected with a VICTOR X4**™** Multilabel Plate Reader (PerkinElmer, Canada). The assay was performed in biological triplicate for each cell variant.

### Adhesion Assay

Cell adhesion to extracellular matrix (ECM) proteins (collagen IV, laminin I and fibronectin) was evaluated using Corning**^®^** BioCoat**™** plates (BD Biosciences, USA). A plate with BSA-coated wells was used as a negative control. The RT-112 cell line variants were seeded in serum-free media at a density of 1.35 × 10^5^ cells per well and incubated for 4** h** at 37°C in 5% CO_2_. Afterwards, the cells were washed three times with 1xPBS and stained with 0.1% crystal violet for 10** min**. After extraction in 10% acetic acid, the absorbance was measured at 560nm with an Infinite F50 Tecan plate reader (Life Sciences, Switzerland). The assay was performed in technical quadruplicate for each cell variant.

### Clonogenic Assay

The RT-112 cell line variants were seeded (1 × 10^3^/well) onto a 6-well plate in full culture medium and incubated for 10 days (37°C, 5% CO_2_). Media was changed every three days. Subsequently, the cells were washed twice with PBS, fixed with 4% paraformaldehyde in PBS solution and stained with 0.005% crystal violet (15 min, RT). The number of colonies was counted using ImageJ software (17). The assay was performed in biological and technical triplicate for each cell variant.

### Wound Healing Assay

The Culture-Insert Well (ibidi GmbH, Germany) was placed into one well of the 24-well plate using sterile tweezers. Cell suspensions were prepared in full medium, then 8 × 10^4^ cells (in volume 100 µl) were transferred to each of four chambers. The cells were incubated at 37°C and 5% CO_2_ to obtain ~100% confluence. After the insert was removed, the cells were gently washed two times with PBS and added to starvation medium. Cell migration was observed by microscope and photographed at intervals (0, 4, 6, 10, 24, 48 h). The area occupied by the cells was determined by TScratch software (18) as % of open image area. The assay was performed in technical quadruplicate for each cell variant.

### Detection of MMP-2 Activity by Gelatin Zymography Assay

RT-112 cell line variants were seeded in full medium on six-well plates and cultured to 80% confluence. The cell starvation medium was collected after 48 h incubation (37°C, 5% CO_2_) and centrifuged. After measuring the protein concentration using Qubit Protein Assay on the Qubit 2.0 Fluorometer (Thermo Fisher Scientific, Netherlands), 3µg of protein was used for SDS-PAGE analysis as described previously (19). The assay was performed in biological triplicate, technical duplicate for each cell variant.

### 3D Culture Growth Assay

Briefly, 1.5 × 10^3^ cells per variant were seeded on a solidified (2mm layer) Geltrex matrix (Thermo Fisher Scientific, Netherlands) and incubated for 19 days in 96-well plate. Geltrex matrix contains a Basement Membrane Matrix including laminin, collagen IV, entactin/nidogen and heparin sulfate proteoglycan, which play an essential role in the tissue organization associated with cell adhesion. After 19 days, the cell lines were observed under the light microscope. This assay was performed in technical triplicate for each cell variant.

### Statistical Analysis

The normality of distribution was determined by the Shapiro-Wilk test; statistical relevance was evaluated with an unpaired t-test. Results with a p-value less than 0.05 were considered as statistically significant.

## Results

### Confirmation of Stable Transductions on the Protein Level

**Figure 1:**
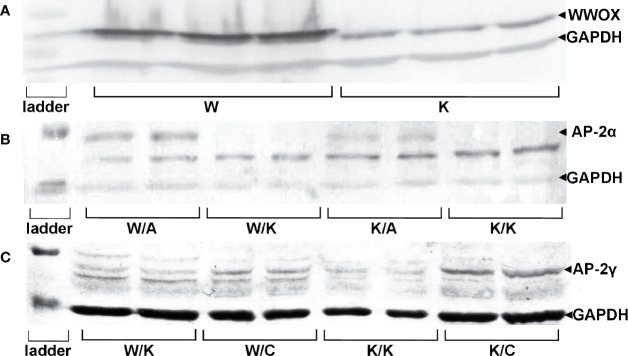
Confirmation of genes overexpression on protein level in specific RT-112 variants. **(A)** WW Domain Containing Oxidoreductase (*WWOX*) overexpression. **(B)** Activating enhancer-binding Protein 2α (AP-2α) overexpression. **(C)** AP-2γ overexpression.

Successful lentiviral transductions were confirmed by determining the relative amount of protein in each variant. This also enabled verification of the WWOX, AP-2α, and AP-2γ proteins levels (**Figure 1**).

The obtained results indicated an increased expression of WWOX in RT-112 WWOX (W) variant (mean expression level 5.10 ± 0.761) compared to RT-112 contr (K) (mean expression level 1.47 ± 0.151, p=0.0013), confirming first stable transduction. Furthermore, the difference between both K/A vs K/K and W/A vs W/K indicated whether second transduction was successful in regard to AP-2α level: mean expression level 1.16 ± 0.136 for K/A vs mean expression level 0.18 ± 0.021 for K/K (p=0.0097) and mean expression level 2.13 ± 0.471 for W/A vs mean expression level 0.09 ± 0.016 for W/K (p=0.0257). The second transduction regarding AP-2γ was confirmed in the same way: expression of AP-2γ in the K/C variant (mean expression level 0.29 ± 0.044) was greater than in K/K (mean expression level 0.06 ± 0.009, p=0.0189) and W/C (mean expression level 0.14, SD ± 0.014) was greater than W/K (mean expression level 0.04 ± 0.013, p=0.0173).

### Cellular Localization of WWOX, AP-2α, and AP-2γ

**Figure 2:**
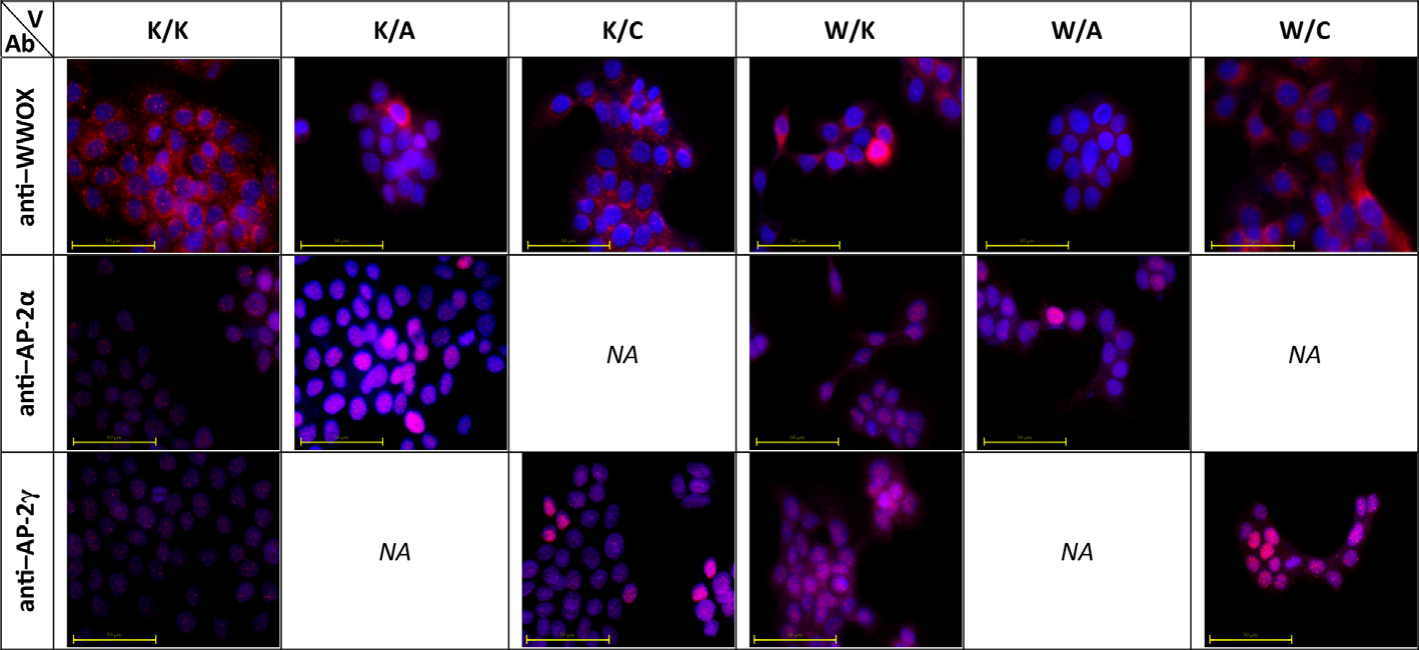
Molecular localization of WW Domain Containing Oxidoreductase (WWOX), activating enhancer-binding Protein 2α (AP-2α), and AP-2γ in developed cellular models. V – variant. Ab – antibody.

Immunofluorescence was performed not only to investigate cellular localization of proteins but also to evaluate whether WWOX capabilities to redistribute AP-2α are similar to that of AP-2γ for which such relationship is already known (11). Pictures taken for immunostaining verification shown that in K/K, K/A, and K/C variants the AP-2α and AP-2γ antibodies appeared to have a nuclear localization while the WWOX antibody was primarily located in the cytoplasmic area but also in the nucleus. In the W/A and W/C variants the anti-WWOX signal originated from the cytoplasm whereas the AP-2 antibodies appeared to be localized both in the cytoplasm and the nucleus. Lastly, in the W/K variant, WWOX, AP-2α, and AP-2γ appeared to be distributed in both cytoplasm and nucleus. These data are visualized in **Figure 2**.

### Assessment of Changes in Mitochondrial Redox Potential, Proliferative Potential and Cellular Apoptosis (Triplex Assay)

**Figure 3:**
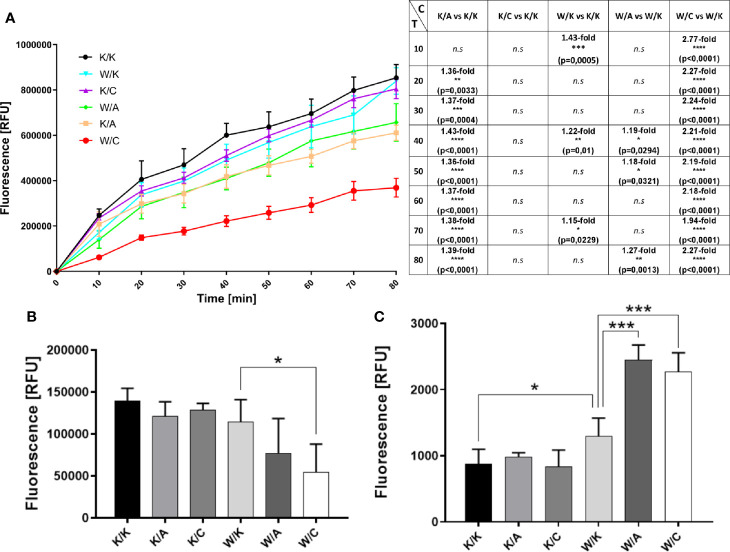
Evaluation of changes in biological processes that affect cancer progression. **(A)** Mitochondrial redox potential (graphical and tabular). C – Comparison. T – Time [min]. **(B)** Proliferation. **(C)** Apoptosis. p < 0.05 (*), p < 0.01 (**), p < 0.001 (***), p < 0.0001 (****).

Redox-dependent phenomena affect cellular processes such as proliferation and apoptosis (20), therefore the use of the Triplex assay allows the assessment of several aspects at once. The role of WWOX in the regulation of respiratory complex and metabolism has already been proposed (21, 22), as well as its impact on apoptosis and proliferation (23). AP-2α and AP-2γ functionality in BLCA is vague yet other research indicated their contrary role in apoptosis and proliferation in other cancer types (24, 25). In the mitochondrial metabolic assessment, all of the five comparisons apart from K/C vs K/K were statistically significant for at least three measurement points (**Figure 3A**). Both W/K and K/A decreased the redox level while K/C did not affect it, all compared to control (K/K). In the case of variants with two genes overexpressed, both W/A and W/C significantly reduced mitochondrial redox potential, with the latter as phenotype having the greatest fold change compared to its control. This suggests that AP‐2γ supervised by WWOX (but not AP-2γ alone) may have a great impact on mitochondrial function and subsequently metabolism. Differences in cell proliferation between variants were determined based on BrdU incorporation during DNA synthesis in dividing cells (**Figure 3B**). The only significant difference (p=0.0317) was a 2-fold decrease of proliferation in W/C compared to its control (W/K). The Triplex assay also indicated a 1.5-fold increase of apoptosis process (p=0.0205) in the W/K variant compared to control (K/K) (**Figure 3C**). Similarly, the same direction of changes was observed for W/A vs W/K and W/C vs W/K comparisons (1.9-fold; p=0.0003 and 1.8-fold; p=0.0005, respectively).

### Estimation of Adhesive Properties

**Figure 4:**
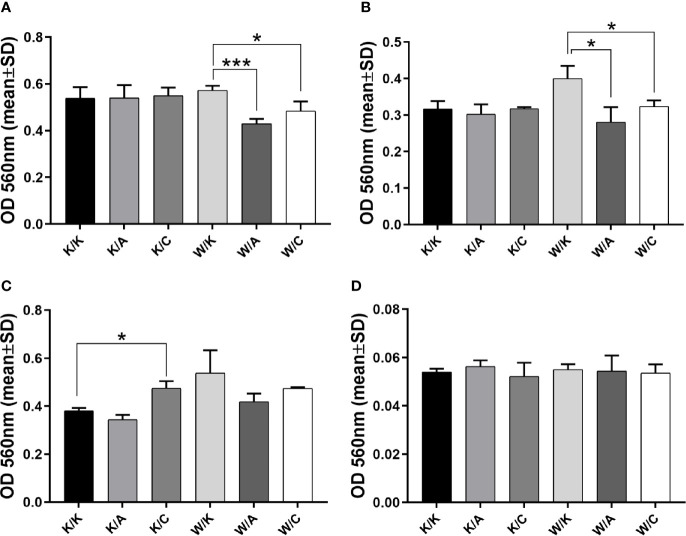
Assessment of adhesive properties to selected extracellular matrix (ECM) proteins. **(A)** Collagen IV. **(B)** Laminin (I) **(C)** Fibronectin. **(D)** BSA. p < 0.05 (*), p < 0.001 (***).

Adhesion assay may not only help to conclude about cellular events but most specifically measure the contacts between cells and ECM proteins (26). Such interaction is not straightforward since in certain cancer types, the binding of cells to the extracellular matrix was proved to enhance the signaling that promoted cell survival, increased proliferation or chemoresistance (27, 28), Thus, the observation of a decrease in cell adhesion to ECM proteins can be interpreted as a phenomenon that indicates a reduction in tumor aggressiveness. This was attributed to BLCA (29), however not in terms of WWOX, AP-2α, or AP-2γ whose function remained unclear. Both W/A and W/C showed a significant decrease in adhesion to collagen IV (p=0.0009 or p=0.028; **Figure 4A**) and laminin I (p=0.0125 or p=0.0139; **Figure 4B**) relative to control variant (W/K); however, the effect was greater for W/A (1.3-fold vs 1.2-fold for collagen IV, and 1.4-fold vs 1.2-fold for laminin I). Similar tendencies for the same comparisons was observed in the adhesion to fibronectin (**Figure 4C**); however, it was not statistically significant. Nevertheless, a statistically significant (p=0.0263) 1.2-fold increase in adhesion to fibronectin was noted for the variant K/C vs K/K. Pure BSA was included as a negative control (**Figure 4D**).

### Evaluation of the Ability of a Single Cell to Grow Into a Colony (Clonogenicity), Migratory Potential and Spatial Structures Growth

**Figure 5:**
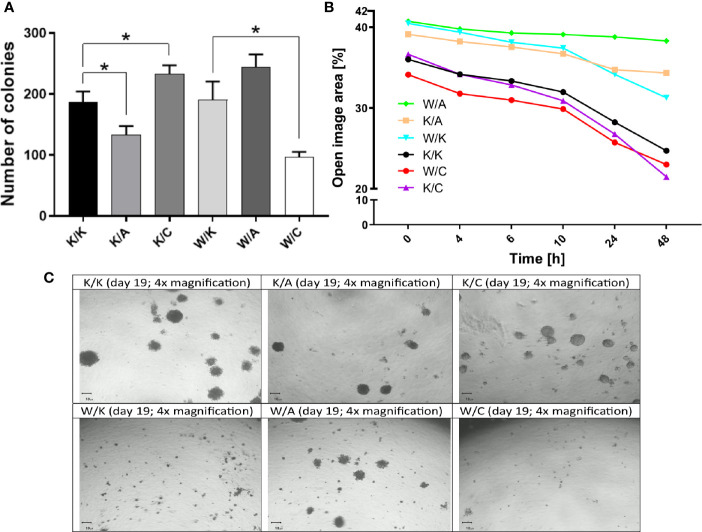
Differences in the number, size and mobility of the bladder cancer colonies. **(A)** Clonogenicity. **(B)** Migratory potential. **(C)** 3D culture growth. p < 0.05 (*).

Assays of colony formation, wound healing and 3D culture growth were performed to systematize function of each cellular variant in terms of self-renewal capacity, cell motility or ability to form tumorspheres, respectively. For instance, WWOX has become involved in the regulation of colony formation (19), as has AP-2α (30). In turn, AP-2γ has been associated with cell motility (25) as well as stemness and chemoresistance (31). Clonogenicity was found to differ depending on the variant considered (**Figure 5A**). First, a statistically significant difference in colony formation capacity was observed compared to control (K/K) for both K/A (1.4-fold decrease; p=0.0168) and K/C (a 1.3-fold increase; p=0.0226). In addition, the W/C variant demonstrated 2.4-fold decrease in clonogenicity compared to its control (W/K) (p=0.0122). Considering wound healing assay, the similarity of the variants to each other, presented in **Figure 5B**, resulted in the establishment of two separate groups. The first group comprised three variants from the top of **Figure 5B** (W/A, K/A, W/K), which were characterized by a <10% change in open image area in terms of difference between the first and the last measurement point. Second group was characterized by a >10% change in open image area, indicating the K/K, K/C, and W/C as variants able to overgrow the wound greater than the other three variants. The 3D culture growth assay (**Figure 5C**) found K/C to generate spatial structures more abundantly than K/K; however, the W/K variant did not produce such clusters although numerous smaller colonies were present. No noticeable differences were observed between K/K and K/A. Compared to W/K, W/A demonstrated larger colonies while W/C demonstrated fewer total colonies.

### Analysis of the Matrix Metalloproteinase Activity

**Figure 6:**
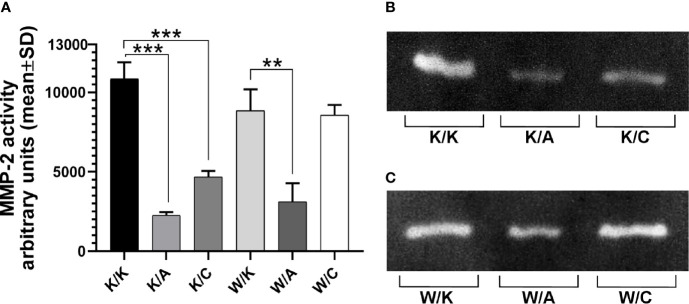
Differences in gelatin zymography. **(A)** Matrix metalloproteinase 2 (MMP-2) active form. **(B)** Gelatin digestion for K/K, K/A, and K/C variants. **(C)** Gelatin digestion for W/K, W/A, and W/C variants. p < 0.01 (**), p < 0.001 (***).

Gelatin zymography was performed in order to investigate invasiveness through matrix metalloproteinase 2 (MMP-2) activity from bladder cancer cells-secreted media of different WWOX/AP-2α/AP-2γ phenotype. A significant decrease in MMP-2 activity was noticed in the case of the K/A or K/C variants relative to their control variant K/K (4.8-fold change; p=0.0002 or 2.3-fold change; p=0.0007, respectively). A statistically significant 2.8-fold decrease in MMP-2 expression was observed in the W/A variant compared to its control (W/K) (p=0.005). These findings are collected in **Figure 6**.

## Discussion

It has been proposed that CFSs may act as cancer-related propulsive factors, as their presence is associated with instability at cytogenetic regions and they often arise prior to carcinogenesis (1, 5). The *WWOX* gene is a non-classical tumor suppressor located at the second most active CFS: FRA16D. Its atypicality results from the fact that the loss of one allele is sufficient to induce further dysfunction of the expressed molecule, which is not suitable to Knudsons’ cancer “two-hit” model and uncommon in the case of tumor suppressor genes (32). Furthermore, this monoallelic scarcity can impact on carcinogenesis due to haploinsufficiency (33).

Currently, literature concerning its involvement in bladder cancer remains limited. However, a significant association is known to exist between progressive loss of *WWOX* expression and increased tumor grade/stage or accelerated cancer progression; this results in a greater chance of an invasive and aggressive status in BLCA (2). The same authors propose that *WWOX* could have the potential to act as a predictive marker in the case of aggressive bladder carcinoma (2). Moreover, tumorigenicity was found to be suppressed based on xenograft mouse model (34). During tumorigenesis of BLCA, *WWOX* expression can be diminished *via* methylation of its promoter region or through loss-of-heterozygosity (LOH) (32). As LOH influence appears to be less important in the regulation of *WWOX* expression in bladder cancer (32), it is possible that epigenetic mechanisms are more relevant; this is supported by the fact that smoking may increase hypermethylation of regions corresponding to *WWOX* exon 1 or its promoter (8).

WWOX can interact with AP-2 family members *via* WW-PPxY interactions (35). In general, both AP-2α and AP-2γ have been implicated in the regulation of cellular proliferation during carcinogenesis (36). Moreover, AP-2γ has been classified as an oncogenic molecule but one that participates in tumor progression rather than initiation (11). The example of Human Epidermal growth factor Receptor 2 (*HER2)* gene as direct target of AP-2γ transcription factor (37, 38) indicates a possible cancer-promoting mechanism of action. The WWOX protein is capable of suppressing the transcriptional activity of oncogenic AP-2γ by sequestering it in the cytoplasm; for example, in the case of breast carcinoma, WWOX inactivation can induce tumor progression by stimulating AP-2γ nuclear translocation (11). Regarding AP-2α, its expression loss during cancer progression directly impairs proto-oncogene c-kit (*c-KIT*) expression and up-regulates Melanoma Cell Adhesion Molecule (*MCAM/MUC18*) (39), but also the thrombin receptor Protease-Activated Receptor-1 (*PAR-1*) and Vascular Endothelial Growth Factor (*VEGF*) genes (40). The fact that these changes are associated with increased tumorigenicity and metastatic abilities clearly indicates that downregulation of AP-2α plays a key role in supporting tumor progression (39).

Although a number of studies have examined the role of WWOX by various aspects, the present study is the first to examine both the role of WWOX with AP-2α and WWOX with AP-2γ through assessment of changes in biological processes on a grade II bladder cancer cell line subjected to two stable lentiviral transductions.

The present study examined the molecular localization of all three proteins of interest using immunocytochemistry. This indicated that the transcription factors AP-2α and AP-2γ remain in the nucleus in the variants with no WWOX overexpression (i.e. K/K, K/A, K/C) but they may become localized in the cytoplasm once WWOX is overexpressed (i.e. W/K, W/A, W/C). These findings are consistent with those of a previous study presenting WWOX protein as a redistributor of AP-2γ to the cytoplasm (11) yet no detailed information is currently available on the interactions between WWOX and AP-2α. Although the AP-2γ PPxY motif has a higher affinity to the WW domain (11), which might explain higher prevalence in the literature compared to AP-2α, it seems both AP-2 proteins are redistributed on the same manner. This is complemented with the fact that WWOX is able to redistribute AP-2 proteins outside the nucleus due to its ability to localize in such compartment (**Figure 2**, visible e.g. in the W/K variant; WWOX antibody).

Since the location of AP-2α/γ can be determined by WWOX, it is likely that the related biological processes will also depend on the expression of each individual. Therefore, a biological assay determining the viability of cancer cells indicated that separate overexpression of WWOX (W/K) or AP-2α (K/A) decreased cell viability, which was not observed during isolated overexpression of AP-2γ (K/C). Similar observations were noticed following WWOX overexpression in colon cancer (41) but also prostate, lung and pancreatic cancers (42), which might be due to enhancement of Tumor Necrosis Factor α (TNFα) cytotoxic function by WWOX (43) where it was confirmed that Reactive Oxygen Species (ROS) are the principal effectors of TNFα-mediated cell death (44). Furthermore, AP-2α viability-suppressing properties were described in hepatocellular carcinoma in case of which cancer cells survival was suppressed *via* inhibition of Extracellular-signal-Regulated Kinase (*ERK*), c-Myc and CyclinD1 (*CCND1*) in Ras-Raf-MEK-ERK pathway (45). The last two are also downstream genes of canonical Wnt signaling pathway which orchestrates survival and self-renewal (46, 47). Lastly, Cluster of Differentiation 133 (*CD133*) downregulation by AP-2α overexpression might suggest independent mechanism focused on suppressing cancer stem cells’ growth and self-renewal (45) which can be crucial for BLCA since CD133 has been proposed as important molecule in terms of bladder cancer viability (48). Regarding the other variants, overexpression of either WWOX and AP-2α (W/A) or WWOX and AP-2γ (W/C) showed an interesting effect. In both K/A and W/K the cell viability was reduced in comparison to their control (K/K); it was similar to their combined form (W/A) which demonstrated a decrease in cell viability compared to control (W/K). This can be accounted for by assuming a synergistic effect between AP-2α and WWOX; however, W/C also demonstrated a dramatic decrease in cell viability while no decrease in tumor cell viability was observed for the variant with separate AP-2γ overexpression (K/C). This suggested that AP-2γ supposedly did not act synergistically with WWOX to cause decrease visible in W/C. We hypothesize that due to the superiority of WWOX over the transcription factor, the presence of oncogene overexpression enhances the protective properties of WWOX aimed at suppressing the tumor progression manifested by AP-2γ.

This is confirmed by the results of the proliferation potential analysis, in which neither WWOX nor AP-2γ reduced cell proliferation when overexpressed separately (W/K or K/C), but a significant reduction in proliferation is observed in the W/C variant. Although no significant decrease in proliferation was observed for the variant with only WWOX overexpression (yet with consequent trend), the literature data indicate that WWOX inhibits proliferation of glioblastoma (49), osteosarcoma (50) and cervical cancer (23). Exemplary scenario based on breast cancer study suggests that it might be explained by WWOX ability to regulate Erb-B2 Receptor Tyrosine Kinase 4 (ErbB4) localization and stability: instead of ErbB4 Intracellular C-terminal Domain (ICD) translocation to the nucleus (which leads to increase in proliferation (51)), full-length receptor is stabilized by WWOX at the cell membrane (9). Together with the aforementioned possibility to sequestrate AP-2γ e.g. to prevent *HER2* expression, this allow to infer that WWOX can regulate proliferation through selected ErbB family receptors, especially since their importance in BLCA tumorigenesis was suggested (52). A mechanism related to Hypoxia-Inducible Factor 1-alpha (HIF1α) has also been proposed, where inactivation of WWOX resulted in the activity of HIF1α and thus its downstream targets, increasing the expression of glycolytic genes and proliferation (53).

Nevertheless, the apoptosis assay is consistent with literature data showing that WWOX induces programmed cell death of bladder cancer, similar to lung and cervical cancer (23, 54). Additionally, an even greater increase was noted in both the W/A and W/C variants, although we believe this similar observation results from different mechanism of action, thus justifying the aforementioned hypothesis. Apparently, AP-2α can promote apoptosis *via* interaction with *MYC* gene based on breast cancer data (55) while AP-2γ is able to downregulate Growth Arrest and DNA-Damage-inducible Beta (*GADD45B*) and Phorbol-12-Myristate-13-Acetate-Induced Protein 1 (*PMAIP1*) in lung cancer e.g. to hinder programmed cell death (25). This might justify their inverse regulation of this process (proving that W/A and W/C phenotypic similarity can be due to different biological actions), yet we did not observe significant changes in apoptosis for separate AP-2α or AP-2γ overexpression in our study. Still, WWOX pro-apoptotic potential may be explained through interaction with p73 protein. During such interplay, p73 sequestration outside nucleus diminishes transcription-dependent apoptosis, yet increases cleavage of p73 in the cytoplasm which then localizes to the mitochondria enhancing TNF-Related Apoptosis-Inducing Ligand (TRAIL)-induced apoptosis (9). This might be of utmost importance as both AP-2α and AP-2γ are able to act synergistically with p73 but Cai *et al*. recently admitted that it is difficult to investigate downstream genes of such interaction, contrary to p53 or p63 (56). Nonetheless, abolishment of AP2-p73 cooperation by WWOX may influence conditions visible in apoptosis assay and is worth considering in future profound investigations.

Subsequent next part of the study estimated the adhesive properties using selected ECM proteins. The only significant difference among variants with single gene overexpression was that the K/C variant (i.e. with separate AP-2γ overexpression) potentiated adhesion to fibronectin compared to K/K. Although these findings cannot be directly compared with previous studies, it has been found that *via* Rho GTPase 3 (*RND3*), AP-2γ regulates extracellular matrix anisotropy i.e. a pathological process of matrix remodeling which occurs during carcinogenesis and where fibronectin is partially implicated (57), indicating its role in generating invasion tracks for cancer cells. The remaining comparisons indicated that adhesion to collagen IV and laminin I was reduced during overexpression of WWOX and AP-2α or WWOX and AP-2γ (W/A or W/C, respectively). A previous study from our department identified that after WWOX silencing, an increase in the expression of integrin α2 and β4 in MFE-296 endometrial cancer cell line was observed (19), thus indicating the possibility of regulating adhesion to collagen and laminin, respectively (58). To the best of our knowledge, there are no scientific reports analyzing changes in the integrin profile regarding AP-2α/γ, while with the use of ELIXIR-approved (59) Integrated System for Motif Activity Response Analysis (ISMARA) tool (60), it was possible to confirm that the α2 integrin-encoding gene is in the top 200 AP-2α targets (based on the deposited project “*Inflammatory response time course, HUVEC (Inoue, 2006)*”; dataset3_v2; ZNF711_TFAP2A_TFAP2D), and both α2 and β4 integrin-encoding genes in the top 200 AP-2γ targets (based on the deposited project “*ENCODE cell lines, expression (Ernst 2011)*”; dataset6.1_ENCODE_expression_v2; TFAP2C). Perhaps the lack of statistical significance for collagen and laminin in the variant with separate overexpression of WWOX results from insufficient effect – apart from guiding AP-2 factors by WWOX, their role in regulating the expression of individual integrins is also needed.

Further tests confirming our hypotheses were focused on evaluation of clonogenicity and spatial structures growth. The former showed that overexpression of AP-2α (K/A) or AP-2γ (K/C) respectively reduced or increased the number of colonies compared to the control (K/K). Similar observations have been noted in research on breast cancer (61) and non-small cell lung cancer (62), where AP-2α re-expression in MDA-MB-231 cells decreased colony formation while AP-2γ knockdown decreased the number of NCI-H292 cell line colonies. Furthermore, despite the overexpression of AP-2γ in both K/C and W/C variants, a completely opposite tendency in clonogenicity is observed suggesting that decreased number of colonies in W/C variant must be due to WWOX overexpression, this being the only difference between K/C and W/C. Such drastic decrease might derive from WWOX attempt to oppose hindrance of apoptosis induced by AP-2γ since WWOX allegedly affects colony formation and cellular growth through Bcl-2/Bax/caspase-3/caspase-9-dependent apoptosis, inducing release of cytochrome c (63). This is not only manifested in apoptosis assay disputed above but can be confirmed by literature data investigating colony formation in cervical cancer (23), myeloma (64) and leukemia (63). Assumption of superiority of such mechanism over AP-2γ ability to increase clonogenicity *via* interaction with Proline-, Glutamate- and Leucine-rich Protein 1 (PELP1) to enhance REarranged during Transfection (RET) signaling (65) is probably one of few explanations yet it fits our study findings. Conclusiveness is also limited due to lack of data regarding PELP1 in bladder cancer but assuming negative correlation between PELP1 and miR-200 family (66) and the fact that these miRNAs are directly repressed by Twist-related protein 1 (TWIST1) in bladder cancer (67) whose expression decreases with the PELP1 knockdown (68), we can assume that its role is not without significance. At last, AP-2α was shown to inhibit DNA synthesis *via* p21^WAF1/CIP1^ activation which affects colony formation (69).

The results of the colony formation test were consistent with those of the 3D culture growth assay which also showed an increase in the amount of spatial structures when AP-2γ was overexpressed. The variant with isolated *WWOX* overexpression was characterized by a smaller number of large spatial colonies, albeit with an increased frequency. Cells overexpressing both WWOX and AP-2γ demonstrated smaller numbers of large spatial structures and reduced formation of colonies, even smaller ones, compared to the control (W/K). An interesting variant is W/A, which presented a marginal decrease in the number of colonies but still formed larger spatial structures. Collectively, it appears that the spheres as clusters with numerous cell growth outward are attributed to AP-2α overexpression which resembles control variant (K/K) while spheres formed after AP-2γ overexpression looked more like the tumorspheres; however, WWOX overexpression suppressed larger spheres formation utterly. What can explain such dissidence is the different role of AP-2γ/AP-2α/WWOX in EMT-MET (Epithelial-to-Mesenchymal Transition and its opposite) which is in close association with stemness potential (70). Namely, AP-2γ may enhance EMT through increased Transforming Growth Factor Beta Receptor 1 (*TGFBR1)* expression which induces p21(RAC1)-Activated Kinase 1 (PAK1) phosphorylation and subsequent Mitogen-Activated Protein Kinase (MAPK) signaling (62) – this is meaningful for BLCA since PAK1 has been related to metastasis and invasion of bladder cancer (71) and MAPK network is itself important in this tumor (72). Moreover, PAK1 is extremely vital for MAPK pathway activation e.g. in colorectal cancer (73) which strengthens the inference on this issue. Additionally, AP-2γ promotes stemness and chemoresistance in the same tumor type although through alterations of different signaling pathway (31). In contrast, AP-2α is more likely to stimulate MET (74) and have activity to shift expression profile toward epithelial phenotype suppressing stem cells properties (75), which might be confirmed by independent experimentally validated AP-2α targets – Insulin-like Growth Factor 1 Receptor (*IGF1R)*, Transportin-1 (*TNPO1)* and Fatty Acid Synthase (*FASN)* – the ones involved in stemness and chemoresistance regulation (76). Furthermore, endogenous AP-2α expression is thought to be downregulated during EMT induced by TGF-β1 (77), proving dissimilarity between AP-2α and AP-2γ in terms of the whole TGF-β network which is crucial for EMT (78). Ultimately, WWOX function relies on EMT inhibition (79) which suggests divergent regulation of this phenotype when it comes to WWOX and AP-2γ. It was proposed this may be due to miRNA modulation e.g. miR-363 and miR-146a with the latter implicated in EMT suppression directly and accumulated *via* negative regulation of c-Myc by WWOX (80). Another exemplary mechanism depends on E74-Like Factor 5/Snail1 (ELF5/Snail1) pathway where WWOX enhance transcriptional activity of ELF5 to inhibit Snail1 and eventually EMT (81). At last, WWOX suppresses stemness based on ovarian and breast cancers (82, 83) which complements our previous research on endometrial cancer showing mesenchymal markers suppression (16).

The only indication that AP-2γ may be able to overcome the effect of WWOX was the fact that the K/C and W/C variants show similar trend in the wound healing assay: in previous assays, they were found to demonstrate opposite trends. Variants with overexpressed AP-2γ alone or with WWOX overexpression, overgrow free space in a similar migratory rate to the cells of the control variant (K/K). However, the remaining variants show analogous propensity i.e. isolated overexpression of AP-2α or WWOX, as well as their combined overexpression slows down the migratory potential. Literature data report that restoration of WWOX expression in osteosarcoma affects e.g. cell motility and that endogenous loss of this gene results in an elevation of Runt-related transcription factor 2 (RUNX2) level, ultimately revealing that direct WWOX-RUNX2 interaction *via* first WW domain of WWOX inhibits RUNX2 function (84, 85). This is important because “macrophage-mediated healing network” (abbreviated “MMHN” by Bagnati *et al.*) correlates with wound healing and is governed by the RUNX2 factor (86). The fact that RUNX2 has been identified as an independent predictor of early tumor recurrence among BLCA patients (87) suggests importance in terms of bladder cancer. Briefly, this would indicate that WWOX can modulate wound healing *via* inhibition of RUNX2-dependent expression and cellular events. How the AP-2γ overcome WWOX control remains enigmatic, yet some data suggest that the AP-2γ also impact on *RUNX2* but using a different, indirect mechanism. It has been documented that the proteins Heart And Neural crest Derivatives-expressed 1 (HAND1) and HAND2 inhibit activation of *RUNX2* (88); of these, the former was found overexpressed during repression of AP-2γ in seminoma (89), but it has been proposed that it may act as a bladder-specific factor (90). In summary, the high expression of AP-2γ might eliminate the repressive effect of HAND1 on *RUNX2* which permits wound healing regulation through the “MMHN” network. In terms of AP-2α, Huang *et al.* concluded that this transcription factor modulates hepatocellular carcinoma cell growth and motility through several pathways i.e. VEGF/Pigment Epithelium-Derived Factor (VEGF/PEDF) signaling pathway mediated by HIF1α, β-catenin/TCF/LEF signaling, mitochondrial pathway depended on Bax/Cytochrome c/Apaf1/Caspase 9, or CdK-inhibitor p21^WAF^ in both p53-dependent and independent pathways (45). Such complex network may have an impact on the final phenotypic effect visible in both variants with AP-2α overexpression.

Finally, gelatin zymography indicated how specific variants regulate MMP-2 activity which can be interpreted in the context of invasive potential of cancer (91, 92). In this experiment, overexpression of AP-2α or AP-2γ inhibited MMP-2 activity where the former reduced it to a greater extent. This was previously demonstrated in glioblastoma, where AP-2α loss correlated with *MMP2* overexpression hence invasion (93). Regarding the variants overexpressing WWOX together with one of two AP-2 transcription factors (i.e. W/A or W/C), the observed decrease in MMP-2 activity (i.e. invasiveness suppression) only in the W/A variant may be dictated predominantly, if not exclusively, by AP-2α overexpression; this would explain the fact that W/K appeared not to have any significant impact on MMP compared to K/K, nor did W/C compared to W/K. The equivalence between the W/K and W/C variants in this regard may confirm the ability of WWOX to have greater influence on the final biological effect than AP-2γ; it was also shown that WWOX did not possess regulatory function on invasiveness properties *via* MMP-2. It is known that the promoter region of the *MMP2* gene contains at least one AP-2 binding element (94). Presumably, the ability to inhibit MMP-2 activity after WWOX and AP-2α overexpression results from greater inhibition of MMP-2 by separate AP-2α overexpression compared to separate AP-2γ overexpression and at the same time increased (as previously mentioned) affinity of AP-2γ PPxY motif to the WW domain of WWOX compared to AP-2α, dictating its further transcriptional activity.

Summarizing each variant individually and assigning putative mechanism of action to specific biological processes, overexpression of AP-2α (K/A) induced a decrease in tumor cell viability (*via* suppression of *ERK* and *CCND1* in Ras-Raf-MEK-ERK and Wnt pathways, or *via CD133* downregulation), migratory potential (through VEGF/PEDF, β-catenin/TCF/LEF and Bax/Cytochrome c/Apaf1/Caspase 9 pathways), MMP-2 activity (hence invasive potential, through direct *MMP2* suppression) and the ability of a single cell to grow into a colony (*via* p21^WAF1/CIP1^ activation). Undoubtedly, the presence of AP-2α has an unfavorable influence on promoting tumor development. Overexpression of AP-2γ (K/C), despite the similarity in regulating the active form of MMP-2 compared to the K/A, resulted in increased capacity for wound growth (by repression of HAND1 to stimulate RUNX2-related manifestation of “MMHN” network), as well as greater adhesion to fibronectin (to guide ECM anisotropy *via* RND3), clonogenicity (enhancing RET signaling *via* interaction with PELP1) and spatial colony formation (increasing *TGFBR1* expression to phosphorylate PAK1 to activate MAPK signaling). This indicates that our findings also confirm available literature data regarding oncogenic nature of AP-2γ. WWOX overexpression (W/K) decreased both cell viability (through TNFα-mediated cell death) and wound overgrowth (*via* inhibition of RUNX2-dependent cellular events e.g. “MMHN”); it also increased apoptosis (enhancing TRAIL-induced apoptosis in mitochondria through p73 sequestration) and reduced the number of large spatial colonies/spheres (through increase in ELF5 transcriptional activity to inhibit Snail1 or *via* negative c-Myc regulation), but with an increase in the frequency of smaller ones. We propose that the *WWOX* gene should be considered as a tumor suppressor in bladder cancer, particularly based on our careful analysis of variants demonstrating overexpression of two genes (W/A or W/C). Overexpression of both WWOX and AP-2α (W/A) reduced the overall number of cultured colonies (but increased the likelihood of higher-order structure formation), induced apoptosis and decreased not only BLCA viability, but also adhesion, invasiveness (based on MMP-2 activity) and migration rate. The nature of this variant apparently results from *tumor suppressor synergism* compared to WWOX or AP-2α overexpression alone: i.e. processes that are not regulated by WWOX itself are manifested by AP-2α and *vice versa*. Finally, WWOX and AP-2γ overexpression (W/C) was associated with not only a decrease in tumor cell viability, proliferation potential, adhesion, clonogenicity and the ability to create spatial structures, but also an increase in apoptosis or intensified migration rate.

Despite the similar endpoint in W/A and W/C, attention on complete inverse regarding separate overexpression of WWOX or AP-2γ is crucial. Briefly, when AP-2γ interacts with overexpressed WWOX, the individual biological processes supporting tumor development are inhibited (compared to K/C). In our opinion, increasing the rate of wound growth in W/C is not a sufficient phenomenon to counteract increased apoptosis and the simultaneous decrease in viability, proliferation, spatial structures formation and clonogenicity. The net effect is that protective biological outcome against cancer can be attributed to WWOX.

To conclude, our findings confirm the previous data indicating the suppressor nature of WWOX but the oncogenic properties of AP-2γ and also shed light on the suppressor characteristics of AP-2α. Co-overexpression of WWOX with AP-2α or WWOX with AP-2γ results in a net anti-tumor effect. However, when considering these findings and the difference between AP-2α and AP-2γ, we suggest that this similarity results from the divergent behavior of WWOX. When WWOX interacts with another tumor suppressor, the nature of its action permits for cooperation; however, when it coexists with an oncogene, there is greater necessity to oppose pro-cancerous actions. Undisputedly, there is still a need to pursue this subject to obtain novel data and to investigate other aspects of bladder cancer.

## Data Availability Statement

The original contributions presented in the study are included in the article/**Supplementary Material**. Further inquiries can be directed to the corresponding author.

## Author Contributions

DK and AKB conceptualized the article. DK, ŻK, AKB, and EP established methodology. DK and ŻK were responsible for software. AKB and EP supervised the article. DK and ŻK visualized the results. DK wrote the original draft. DK, ŻK, and EP reviewed and edited the article.

## Funding

This research was supported by the National Science Centre of Poland, grant number 2016/21/B/NZ2/01823.

## Conflict of Interest

The authors declare that the research was conducted in the absence of any commercial or financial relationships that could be construed as a potential conflict of interest.

## Abbreviations

AP-2, Activating enhancer-binding Protein 2; BLCA, Bladder cancer; BrdU, 5-bromo-2′-deoxyuridine; CCND1, CyclinD1; CD133, Cluster of Differentiation 133; CFS, Common fragile site; c-KIT, Proto-oncogene c-kit; DSMZ, Deutsche Sammlung von Mikroorganismen und Zellkulturen; ECM, Extracellular matrix; ELF5, E74-Like Factor 5; EMT, Epithelial-to-Mesenchymal Transition; ErbB4, Erb-B2 Receptor Tyrosine Kinase 4; ERK, Extracellular-signal-Regulated Kinase; FASN, Fatty Acid Synthase; FHIT, Fragile Histidine Triad Diadenosine Triphosphatase; GADD45, Growth Arrest and DNA-Damage-inducible Beta; HAND1, Heart And Neural crest Derivatives-expressed 1; HER2, Human Epidermal growth factor Receptor 2; HIF1α, Hypoxia-Inducible Factor 1-alpha; ICD, Intracellular C-terminal Domain; IGF1R, Insulin-like Growth Factor 1 Receptor; ISMARA, Integrated System for Motif Activity Response Analysis; LOH, Loss-of-heterozygosity; MAPK, Mitogen-Activated Protein Kinase; MCAM, Melanoma Cell Adhesion Molecule; MET, Mesenchymal-to-Epithelial Transition; MMHN, macrophage-mediated healing network; MMP-2, Matrix Metalloproteinase 2; OS, Overall survival; PAK1, p21(RAC1)-Activated Kinase 1; PARK2, Parkinson disease-2; PAR-1, Thrombin receptor Protease-Activated Receptor-1; PEDF, Pigment Epithelium-Derived Factor; PELP1, Proline-, Glutamate- and Leucine-rich Protein 1; PFS, Progression-free survival; PMAIP1, Phorbol-12-Myristate-13-Acetate-Induced Protein 1; PMSF, Phenylmethylsulfonyl fluoride; PPxY, Proline-rich motif; RET, REarranged during Transfection; RND3, Rho GTPase 3; ROS, Reactive Oxygen Species; RUNX2, Runt-related transcription factor 2; TFAP2, Transcription factor AP-2; TGFBR1, Transforming Growth Factor Beta Receptor 1; TNFα, Tumor Necrosis Factor α; TNPO1, Transportin-1; TRAIL, TNF-Related Apoptosis-Inducing Ligand; TWIST1, Twist-related protein 1; VEGF, Vascular Endothelial Growth Factor; WWOX, WW Domain Containing Oxidoreductase.
