# Corrigendum: Fragile Gene *WWOX* Guides *TFAP2A*/*TFAP2C*-Dependent Actions Against Tumor Progression in Grade II Bladder Cancer

**DOI:** 10.3389/fonc.2021.735435

**Published:** 2021-08-26

**Authors:** Damian Kołat, Żaneta Kałuzińska, Andrzej K. Bednarek, Elżbieta Płuciennik

**Affiliations:** Department of Molecular Carcinogenesis, Medical University of Lodz, Łódź, Poland

**Keywords:** bladder cancer, WWOX, TFAP2A, TFAP2C, AP-2alpha, AP-2gamma

Andrzej K. Bednarek was not included as an author in the published article. The corrected Author Contributions Statement appears below.

The authors apologize for this error and state that this does not change the scientific conclusions of the article in any way. The original article has been updated.

## Author Contributions

DK and AKB conceptualized the article. DK, ŻK, AKB, and EP established methodology. DK and ŻK were responsible for software. AKB and EP supervised the article. DK and ŻK visualized the results. DK wrote the original draft. DK, ŻK, and EP reviewed and edited the article.

## Publisher’s Note

All claims expressed in this article are solely those of the authors and do not necessarily represent those of their affiliated organizations, or those of the publisher, the editors and the reviewers. Any product that may be evaluated in this article, or claim that may be made by its manufacturer, is not guaranteed or endorsed by the publisher.

