# Supplementary material for: Fragile Gene WWOX Guides TFAP2A/TFAP2C-Dependent Actions Against Tumor Progression in Grade II Bladder Cancer
Source: Front Oncol. 2021 Feb 25;11:621060. doi: 10.3389/fonc.2021.621060 (PMC7947623; doi:10.3389/fonc.2021.621060)
Supplement: Supplementary file 1 [file DataSheet_1.docx]

Supplementary Material

# Supplementary Figures

**

**

**Supplementary Figure 1.** Diagram showing the double transduction scheme of RT-112 cell line.
